# LncRNA TRPM2-AS promotes ovarian cancer progression and cisplatin resistance by sponging miR-138-5p to release SDC3 mRNA

**DOI:** 10.18632/aging.202541

**Published:** 2021-02-17

**Authors:** Yi Ding, Xiangyu Tan, Abuduyilimu Abasi, Yun Dai, Ruxing Wu, Tao Zhang, Kexin Li, Miao Yan, Xiaoyuan Huang

**Affiliations:** 1 Department of Obstetrics and Gynecology, Cancer Biology Research Center, Tongji Hospital, Tongji Medical College, Huazhong University of Science and Technology, Wuhan 430030, Hubei, P.R. China

**Keywords:** lncRNAs, TRPM2-AS, miR-138-5p, SDC3, ovarian cancer

## Abstract

The role of TRPM2-AS lncRNA in OvC has not been explored. This study aimed to investigate whether and how TRPM2-AS contributes to the progression of OvC. First, qRT-PCR was employed to measure the expression of TRPM2-AS, miR-138-5p and *SDC3* in OvC samples. A xenograft formation assay was subsequently performed to detect the tumor growth *in vivo*. The cell viability, colony formation, cell migration, cell invasion and cell apoptosis were later evaluated using a series of experiments. The western blot assay was utilized to detect the *SDC3* protein expression and cell-apoptosis markers. Luciferase reporter gene assay, RIP, and RNA pull-down assays were performed to identify the association between TRPM2-AS, miR-138-5p and *SDC3*. Findings indicated that the expression of TRPM2-AS and *SDC3* was significantly upregulated in OvC tissues and cells, while miR-138-5p expression was significantly downregulated in OvC samples. Unlike miR-138-5p, TRPM2-AS and *SDC3* were found to promote OvC development. It was also found that TRPM2-AS could sponge miR-138-5p to release *SDC3*, thus promoting OvC progression. Apart from that, we discovered that both sh-TRPM2-AS and cisplatin could enhance the apoptosis of OvC cells. Overall, our findings suggested that the TRPM2-AS/miR-138-5p/*SDC3* axis was closely associated with OvC tumorigenesis and cisplatin resistance.

## INTRODUCTION

Ovarian cancer (OvC) is one of the most common gynecological malignancies, threatening women’s health due to its high incidence and high mortality rate [1]. This cancer is highly fatal, and OvC can be treated effectively using aggressive surgical debulking and chemotherapy [2]. Cisplatin, a chemotherapy drug, induces cell apoptosis and prevents the spread of OvC [3]. However, cisplatin resistance greatly attenuates the anti-cancer effect of cisplatin on OvC [4]. Therefore, it is urgent to unravel the molecular mechanism of cisplatin resistance to facilitate the diagnosis and treatment of OvC.

Long non-coding RNAs (lncRNAs) have more than 200 nucleotides, and they have been reported to participate in the progression of cancers with cisplatin resistance [5–9]. For instance, one research on OvC showed that lncRNA UCA1 was upregulated in cisplatin-resistant tissues and cells and that it induced cisplatin resistance by regulating the miR-143/FOSL2 axis [10]. LncRNA EBIC was also reported to promote OvC cells’ cisplatin resistance by regulating the Wnt/β-catenin pathway [11]. LncRNA TRPM2-AS is an antisense RNA of TRPM2 that induces cell death under oxidative stress [12]. It has been found to play tumor-promotive roles in multiple cancers such as breast cancer [13], gastric cancer [14], and colorectal cancer [15]. In 2017, Ma et al. [16] discovered that TRPM2-AS knockdown attenuated the cisplatin resistance of non-small cell lung cancer. However, the function of TRPM2-AS in OvC progression and cisplatin resistance has not been explored.

MicroRNAs (miRNAs) have attracted the attention of researchers interested in the treatment and development of cancers [17]. Previous studies demonstrated that miRNAs acted as an oncogene or anti-oncogene in OvC. For instance, miR-126-3p targeted PLXNB2 and suppressed the proliferation and invasion of OvC cells [18]. In another experiment, miR-590-3p promoted the xenograft formation and metastasis of OvC cells *in vivo* [19]. Also observed was the negative effect of miR-138 on OvC tumorigenesis [20]. A member of miR-138, miR-138-5p was discovered to be downregulated in cisplatin-resistant cells and was found to enhance the cisplatin sensitivity of OvC cells [21]. Nevertheless, the regulatory mechanism of miR-138-5p in cisplatin resistance of OvC cells remains unclear.

*Syndecan 3* (*SDC3*) encodes a protein belonging to the syndecan proteoglycan family, which plays a role in cell shape organization. Mainly distributed in neural tissues and responsible for developing musculoskeletal tissues [22], SDC3 was demonstrated to overexpress and mediate the metastasis of prostate cancer [23]. Another study showed that SDC3 regulated by the circSCARB1/miR-510-5p axis functioned as an oncogene in renal cell carcinoma [24]. However, the function of SDC3 in OvC has not been thoroughly explored.

In this study, our aim was to explore not only the role of the TRPM2-AS/miR-138-5p/SDC3 axis in OvC but also the effect of TRPM2-AS on OvC cells with cisplatin resistance. Our findings could assist not only in providing a novel molecular mechanism for the cisplatin resistance of OvC but also in improving OvC treatments.

## RESULTS

### TRPM2-AS promoted OvC progression *in vitro*

**Figure 1:**
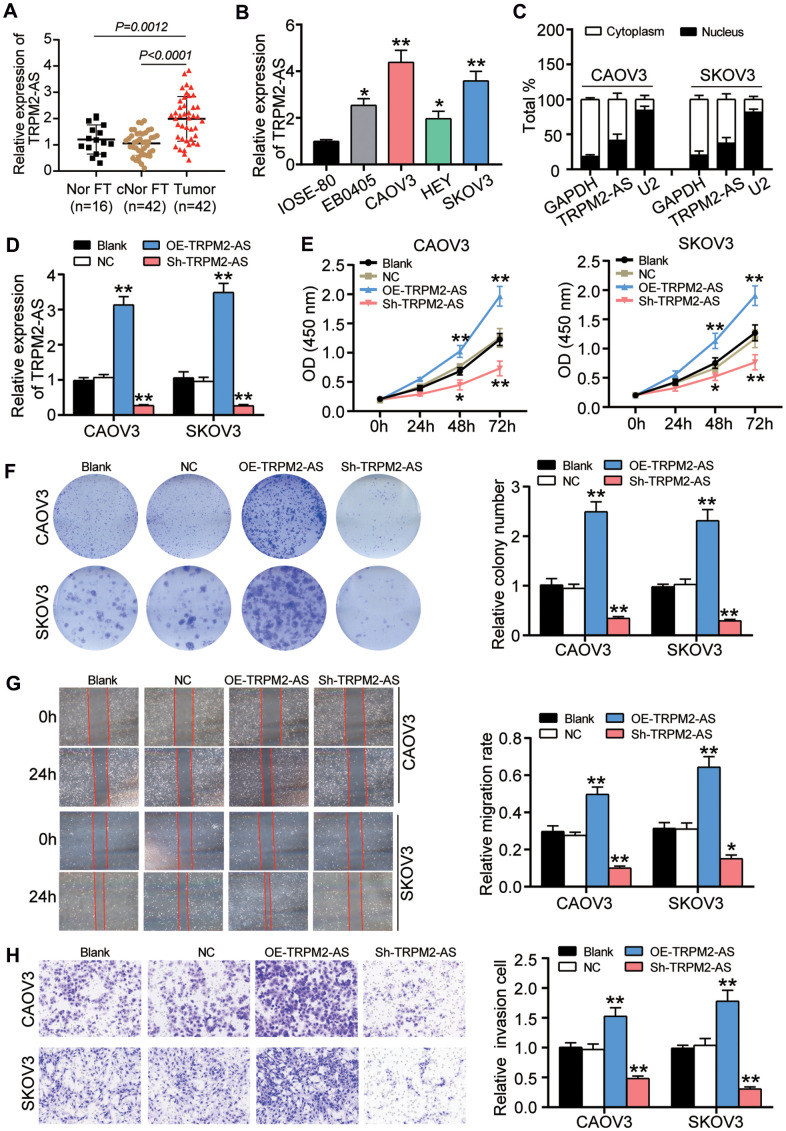
**TRPM2-AS contributed to the OvC progression *in vitro*.** (**A**) RT-qPCR analysis revealed that the TRPM2-AS was overexpressed in ovarian tumor tissues (Tumor) compared with the contralateral normal fallopian tube tissues (cNor FT) and normal fallopian tube tissues from patients with benign gynecological tumor (Nor FT). (**B**) RT-qPCR analysis revealed that the TRPM2-AS was overexpressed in four OvC cell lines (EB0405, CAOV3, HEY and SKOV3) compared with human ovarian surface epithelial cell line IOSE-80. (**C**) TRPM2-AS was mainly located in cytoplasm. (**D**) The high transfection efficiency of sh-TRPM2-AS and TRPM2-AS overexpression in CAOV3 and SKOV3 cells. (**E**) TRPM2-AS was proved to promote cell viability in CAOV3 and SKOV3 cells by CCK8 assay. (**F**) The colony formation ability was enhanced by TRPM2-AS. (**G**) TRPM2-AS promoted cell migration by the assessment of wound healing assay. (**H**) TRPM2-AS enhanced the cell invasive ability by Transwell invasion assay. Blank, blank control. NC, negative control. OE-TRPM2-AS, TRPM2-AS overexpression vectors. Sh-TRPM2-AS, TRPM2-AS knockdown vectors. *P<0.05, **P<0.001, compared with the blank group.

The expression of TRPM2-AS was first detected in OvC tissues and cells to identify the role of TRPM2-AS in OvC. Given the fallopian tube was the most important origin of OvC [25, 26], we detected the expression of TRPM2-AS in 42 OvC tissue samples and the paired contralateral fallopian tube tissues. Our experimental results demonstrated that the expression of TRPM2-AS in OvC tissues increased by 2-fold compared to that in the contralateral normal fallopian tube tissues. As for the normal fallopian tube tissues from 16 patients with benign gynecological tumors that were collected to also serve as the control, we found a significantly lower TRPM2-AS expression in contrast to OvC tissues (Figure 1A). Similarly, the expression of TRPM2-AS was found to be upregulated in four OvC cell lines (EB0405, CAOV3, HEY and SKOV3). Compared to IOSE-80 cell line (human ovarian surface epithelial cells), the TRPM2-AS with more than 3-fold upregulation was observed in CAOV3 and SKOV3 cells (Figure 1B). Hence, CAOV3 and SKOV3 cells were selected to perform subsequent experiments, and both of the two OvC cell lines derives from the patients with high-grade serous carcinoma (HGSC), which is the most common subtype of ovarian cancer. After analyzing the results of nuclear and cytoplasmic fractionation and qRT-PCR, we observed that TRPM2-AS was mainly distributed in the cell cytoplasm (Figure 1C). The sh-TRPM2-AS (TRPM2-AS shRNA) and OE-TRPM2-AS (TRPM2-AS overexpression) vectors transfection respectively led to significantly downregulated and upregulated TRPM2-AS in CAOV3 and SKOV3 cells, suggesting the efficient transfection (Figure 1D). Our findings also revealed that the viability of the OE-TRPM2-AS group was enhanced at 48 and 72 hours, while sh-TRPM2-AS impaired the viability of both CAOV3 and SKOV3 cell lines (Figure 1E). The colony formation assay results indicated that the colony-forming ability was enhanced by more than 50% by OE-TRPM2-AS transfection, whereas sh-TRPM2-AS impaired the colony-forming ability by about 60% in both OvC cell lines (Figure 1F). We observed a similar trend in the wound-healing assay results, which showed that TRPM2-AS overexpression elevated the migration rate by 1.7-fold in CAOV3 cells and 2.1-fold in SKOV3 cells but that TRPM2-AS silence impeded the migration rate (Figure 1G). Finally, we noticed that the cell-invasive capability was enhanced by more than 50% in the OE-TRPM2-AS group, while a 50% decrease in the cell invasion capability was observed in the sh-TRPM2-AS group (Figure 1H).

### Silencing TRPM2-AS inhibited tumor growth *in vivo*

**Figure 2:**
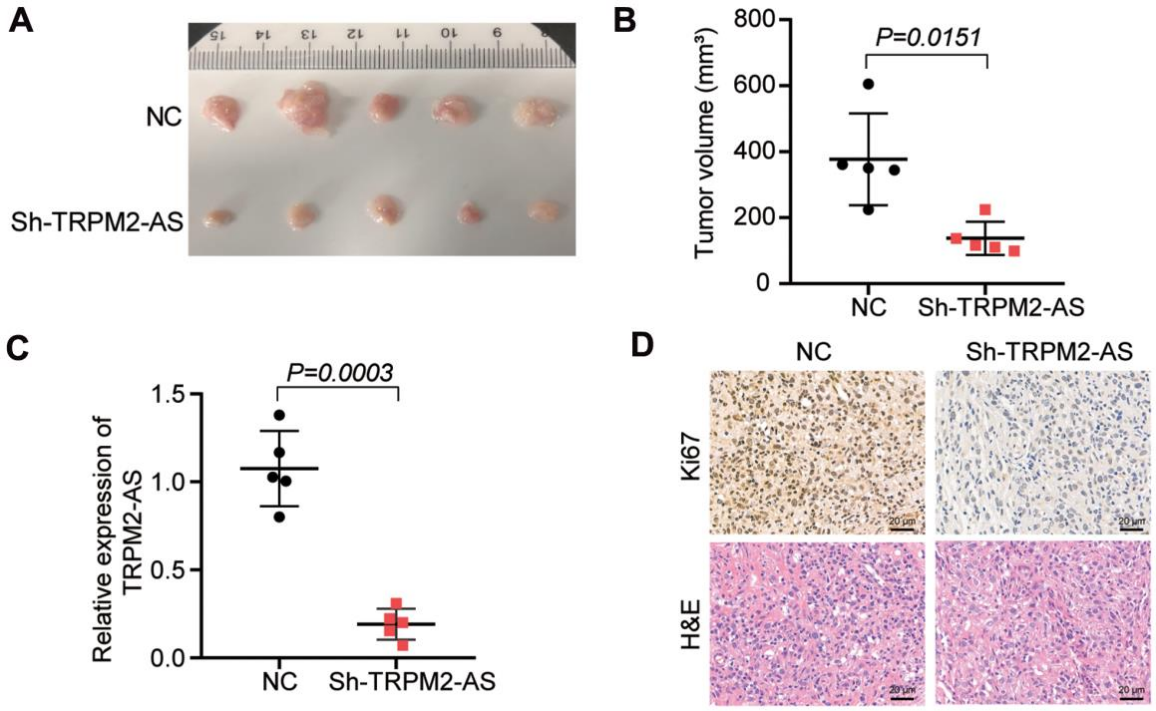
**TRPM2-AS knockdown inhibited the OvC progression *in vivo*.** (**A**) The subcutaneous tumors derived from the mice with the negative control transfected SKOV3 cells or sh-TRPM2-AS transfected SKOV3 cells for 30 days. (**B**) The tumor volumes were measured 30 days after the negative control or sh-TRPM2-AS transfected SKOV3 cells were subcutaneously injected into the nude mice. (**C**) The TRPM2-AS expression in the tumor tissues. (**D**) Ki67 staining and H&E pathological staining assay proved the inhibitory effect of TRPM2-AS knockdown on cell proliferation *in vivo*. Scale bar, 20 μm. Sh-TRPM2-AS, TRPM2-AS knockdown vectors. **P<0.001.

The effect of TRPM2-AS on tumor growth *in vivo* was explored in nude mice subcutaneously injected with the sh-TRPM2-AS transfected SKOV3 cells. According to the results, the tumors derived from the sh-TRPM2-AS group were smaller than those from the NC group after injecting the nude mice for 30 days (Figure 2A). The tumor volume in the sh-TRPM2-AS group was reduced by more than 50% compared to the NC group (Figure 2B). The qRT-PCR outcome displayed that TRPM2-AS expression was downregulated by 80% in the tumors from the nude mice subcutaneously injected with the sh-TRPM2-AS transfected SKOV3 cells (Figure 2C). After performing the Ki67 staining assay and H&E pathological staining, we found that TRPM2-AS knockdown inhibited Ki67 expression and tumor aggravation *in vivo* (Figure 2D). This result suggested that sh-TRPM2-AS inhibited tumor proliferation *in vivo*.

### miR-138-5p regulated TRPM2-AS

**Figure 3:**
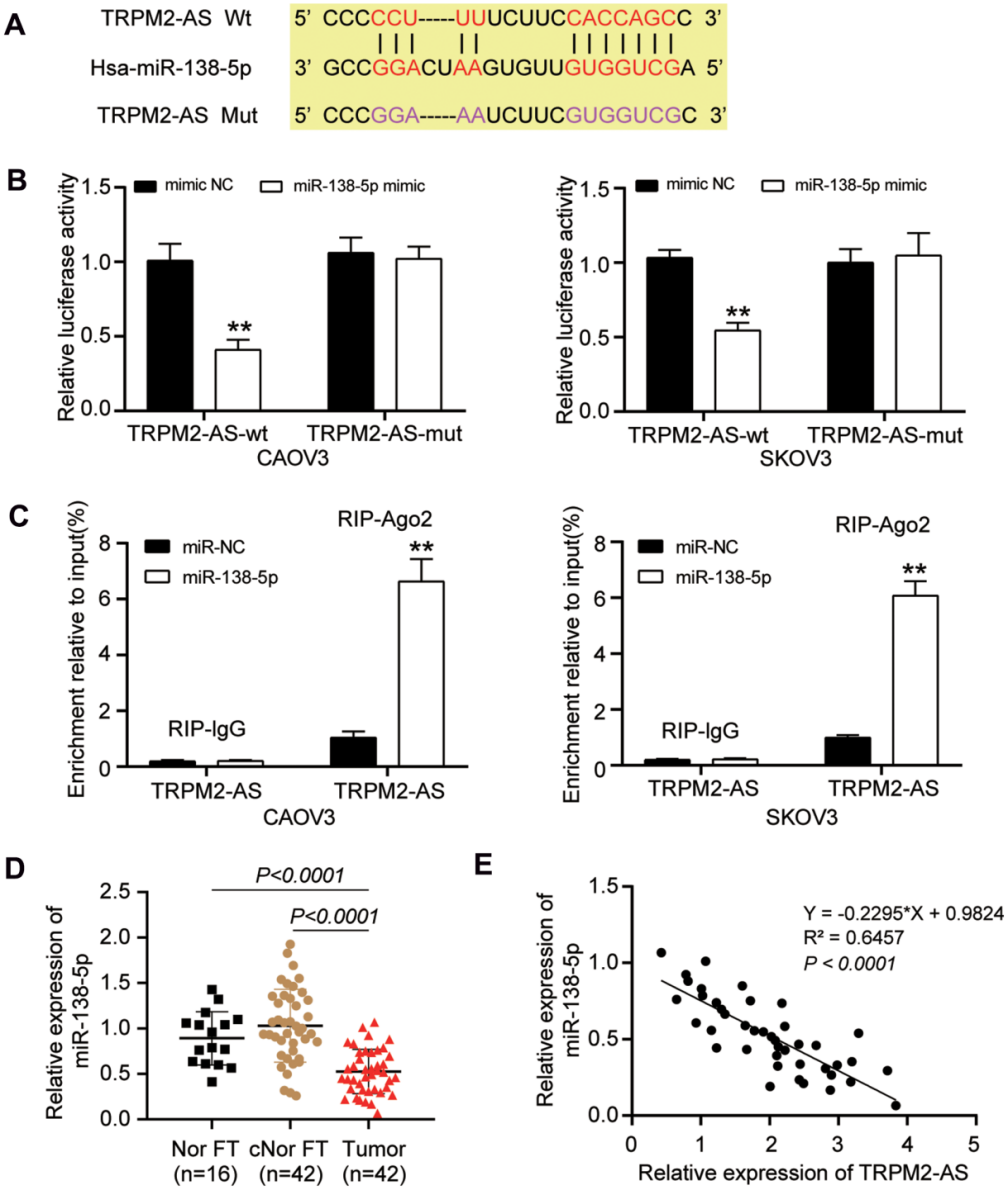
**TRPM2-AS could sponge miR-138-5p**. (**A**) ENCORI starBase predicted the binding site between TRPM2-AS and miR-138-5p. (**B**) The TRPM2-AS could sponge miR-138-5p, validated by luciferase assay. TRPM2-AS-wt, wild-type TRPM2-AS containing the binding site. TRPM2-AS-mut, mutant TRPM2-AS without the binding site. (**C**) RIP assay further proved the binding site between TRPM2-AS and miR-138-5p. (**D**) RT-qPCR analysis revealed that the miR-138-5p expression was downregulated in OvC tissues compared with contralateral normal fallopian tube tissues (cNor FT) and normal fallopian tube tissues from patients with benign gynecological tumor (Nor FT). (**E**) The expression of TRPM2-AS and miR-138-5p was negatively correlated. **P<0.001.

The binding site between TRPM2-AS and miR-138-5p was predicted using the ENCORI starBase system. The wild-type (WT) TRPM2-AS contained the binding site, while the binding site was mutated from “CCUUU-----CACCAGC” to “GGAAA-----GUGGUCG” as the mutant (Mut) TRPM2-AS (Figure 3A). The luciferase reporter assay result showed a 50% decrease in the luciferase activity in the cells co-transfected with TRPM2-AS-WT and miR-138-5p mimic; however, TRPM2-AS-Mut could not affect the luciferase activity in CAOV3 and SKOV3 cells (Figure 3B). The RIP assay results further validated the binding site between TRPM2-AS and miR-138-5p (Figure 3C). Apart from that, the expression of miR-138-5p was measured in 42 paired ovarian tumor tissues and contralateral normal fallopian tube tissues, and 16 normal fallopian tube tissues from patients with benign gynecological tumors. The result displayed that the miR-138-5p was downregulated by nearly 50% in ovarian tumor tissues in contrast to both the contralateral normal fallopian tube tissues and the normal fallopian tube tissues from patients with benign gynecological tumors (Figure 3D). Besides, the Pearson correlation analysis showed that TRPM2-AS expression was negatively correlated with miR-138-5p expression in OvC tumor tissues (Figure 3E).

### miR-138-5p reversed the effect of TRPM2-AS on OvC cells

**Figure 4:**
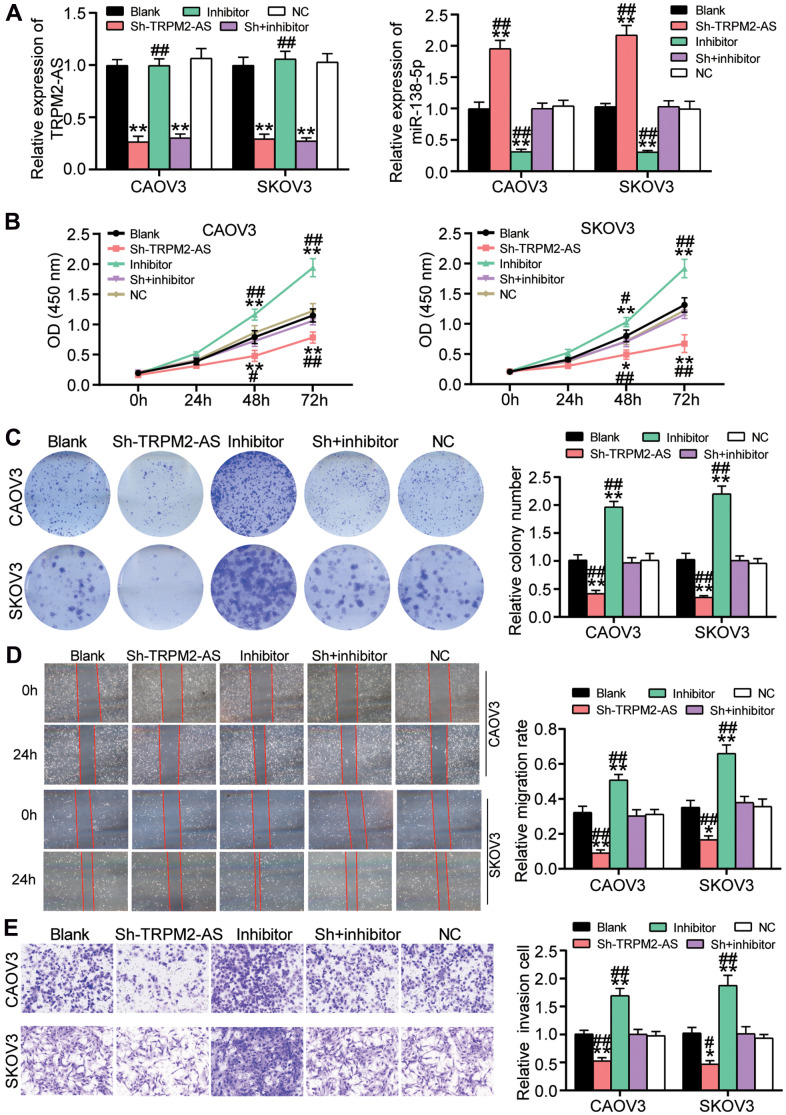
**The inhibitory effect of TRPM2-AS knockdown on OvC progression was attenuated by miR-138-5p inhibitor.** (**A**) The high transfection efficiency of sh-TRPM2-AS and miR-138-5p inhibitor in CAOV3 and SKOV3 cells. (**B**) MiR-138-5p inhibitor attenuated the inhibitory effect of TRPM2-AS knockdown on cell viability in CAOV3 and SKOV3 cells. (**C**) The inhibitory effect of TRPM2-AS knockdown on colony formation was overturned by miR-138-5p inhibitor. (**D**) The negative role of sh-TRPM2-AS on cell migration was relieved by miR-138-5p inhibitor. (**E**) The negative role of sh-TRPM2-AS on cell invasion was impaired by miR-138-5p inhibitor. Blank, blank control. NC, negative control. Sh, Sh-TRPM2-AS. Inhibitor, miR-138-5p inhibitor. *P<0.05, **P<0.001 compared with blank control. ^##^*P*<0.001 compared with co-transfection of sh-TRPM2-AS and miR-138-5p inhibitor.

Because of the negative correlation between TRPM2-AS and miR-138-5p, the function of the TRPM2-AS/miR-138-5p axis in OvC cells was further explored. Using qRT-PCR, the transfection efficiency of sh- TRPM2-AS and miR-138-5p inhibitor was validated in CAOV3 and SKOV3 cells. The result showed that sh-TRPM2-AS reduced TRPM2-AS expression by 70% and elevated miR-138-5p expression by 2-fold. Even though miR-138-5p inhibitor downregulated miR-138-5p by 70%, it had no effect on TRPM2-AS expression (Figure 4A). As shown in Figure 4B, the miR-138-5p inhibitor enhanced the viability of CAOV3 and SKOV3 cells. Nonetheless, co-transfecting the cells with sh-TRPM2-AS attenuated the promotive role of the miR-138-5p inhibitor on cell viability. The colony formation assay results displayed that the number of colonies in the cells transfected with the miR-138-5p inhibitor was twice that of the blank control cells. However, the number of colonies in the cells co-transfected with sh-TRPM2-AS and the miR-138-5p inhibitor was similar to that of the blank control cells (Figure 4C). Similar to the colony formation assay findings, the wound-healing assay results revealed that the miR-138-5p inhibitor not only promoted cell migration but also reversed the inhibitory effect of sh-TRPM2-AS on cell migration (Figure 4D). Besides, the miR-138-5p inhibitor increased cell invasion by 50% and impaired the inhibitory effect of sh-TRPM2-AS on cell invasion (Figure 4E).

### Relationship between SDC3 and miR-138-5p

**Figure 5:**
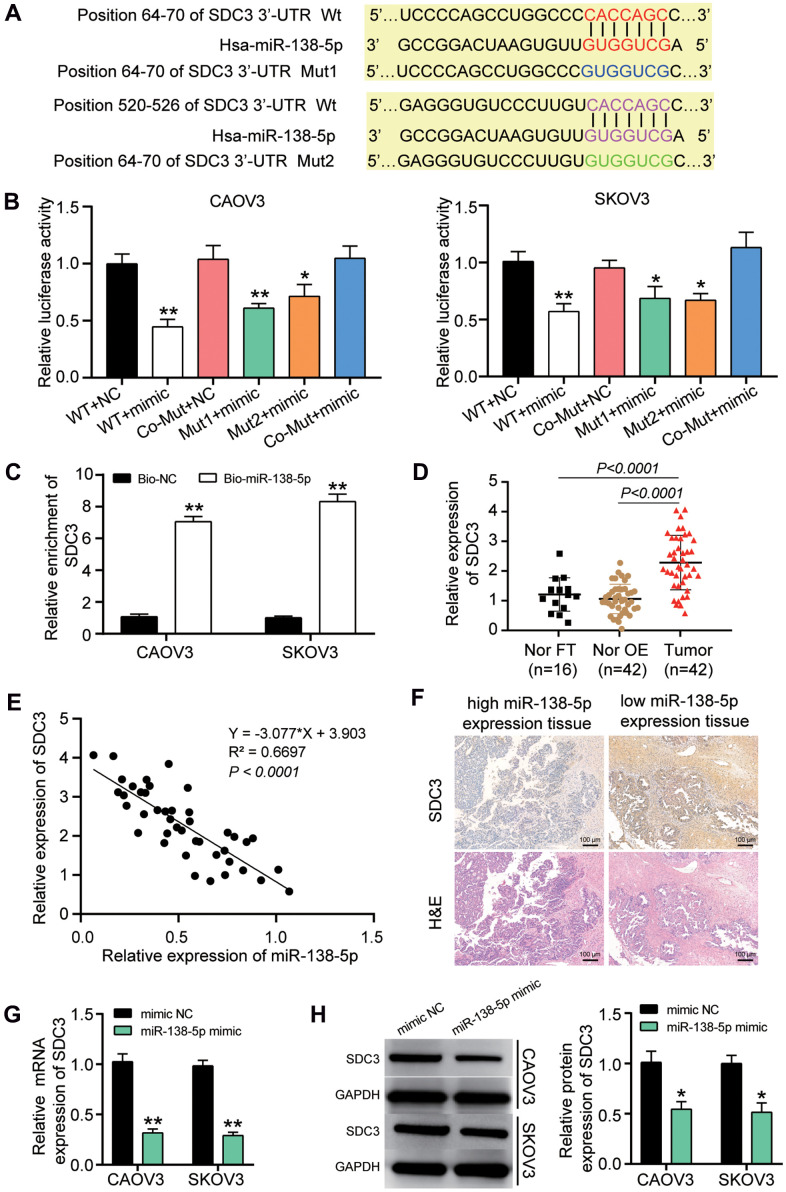
**miR-138-5p could target SDC3 in OvC cells.** (**A**) TargetScan predicted the two binding sites between miR-138-5p and SDC3. (**B**) SDC3 was proved to be targeted by miR-138-5p using luciferase assay. WT, wild-type SDC3 3’UTR containing the two binding sites. Mut1, mutant SDC3 mRNA 3’UTR without the binding site 1. Mut2, mutant SDC3 mRNA 3’UTR without the binding site 2. Co-Mut, mutant SDC3 3’UTR without the two binding sites. NC, negative control. Mimic, miR-138-5p mimic. (**C**) RNA pull-down assay further proved the binding site between SDC3 mRNA 3’UTR and miR-138-5p. Bio-NC, biotin-labeled negative control. Bio-miR-138-5p, biotin-labeled miR-138-5p mimic. (**D**) RT-qPCR analysis revealed that the SDC3 mRNA expression was upregulated in OvC tissues compared with contralateral normal fallopian tube tissues (cNor FT) and normal fallopian tube tissues from patients with benign gynecological tumor (Nor FT). (**E**) The negative correlation between SDC3 mRNA expression and miR-138-5p in OvC tissues. (**F**) IHC assay showed the low-level SDC3 in high-level miR-138-5p tissues. H&E pathological staining was performed to indicate the ovarian tumor type. The tumor type in all images were identified as high-grade serous carcinoma (HGSC), while the tissues in left and right column images was respectively 1c sub-stage and 3c sub-stage. Scale bar, 100 μm. (**G**) The expression of SDC3 mRNA decreased in CAOV3 and SKOV3 cells with the transfection of miR-138-5p mimic. (**H**) The expression of SDC3 protein reduced in CAOV3 and SKOV3 cells with the transfection of miR-138-5p mimic. **P<0.001.

TargetScan was employed to predict the two binding sites of SDC3 3’UTR for miR-138-5p (Figure 5A). The two binding sites on SDC3 mRNA 3’UTR were first mutated before performing luciferase assay. The luciferase reporter assay results showed that the lowest luciferase activity (a 50% decrease) was observed when the wild-type SDC3 mRNA 3’UTR and miR-138-5p mimic was co-transfected (Figure 5B). The RNA pull-down assay analysis further proved that the SDC3 mRNA could be pulled down when the cells were transfected with the biotin-labeled miR-138-5p mimic (Figure 5C). In our clinical specimens, the SDC3 expression was upregulated by approximately 2-fold in OvC tissues compared to both contralateral normal fallopian tube tissues and normal fallopian tube tissues from patients with benign gynecological tumors (Figure 5D). The subsequent correlation analysis revealed that the SDC3 mRNA expression was negatively correlated with miR-138-5p expression in OvC tissues (Figure 5E). The IHC assay result further confirmed that while the expression of SDC3 was high in the ovarian tumor tissues with lower expression of miR-138-5p, the expression of SDC3 was low in the ovarian tumor tissues with higher expression of miR-138-5p (Figure 5F). In CAOV3 and SKOV3 cells, the expression of SDC3 mRNA and protein was reduced by 75% and 50%, respectively, in the miR-138-5p mimic transfection group (Figure 5G, 5H).

### SDC3 attenuated the inhibitory effect of miR-138-5p in OvC cells

**Figure 6:**
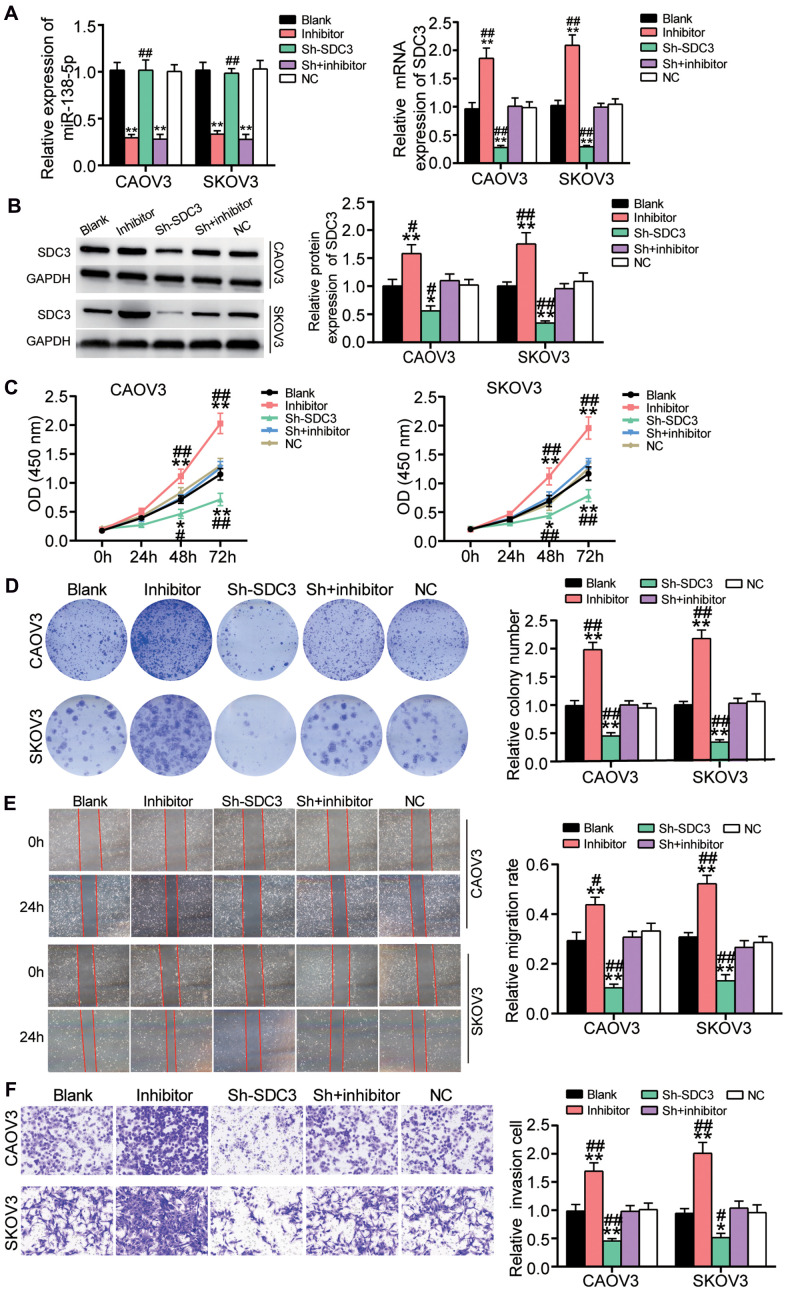
**The positive effect of miR-138-5p inhibitor on OvC progression was relieved by SDC3 silence.** (**A**) The high transfection efficiency of sh-SDC3 and miR-138-5p inhibitor was detected by qRT-PCR in CAOV3 and SKOV3 cells. (**B**) The high transfection efficiency of sh-SDC3 and miR-138-5p inhibitor was further measured by western blot analysis in CAOV3 and SKOV3 cells. (**C**) sh-SDC3 attenuated the promotive effect of miR-138-5p inhibitor on cell viability in CAOV3 and SKOV3 cells. (**D**) The positive effect of miR-138-5p inhibitor on colony formation was overturned by sh-SDC3. (**E**) The positive role of miR-138-5p inhibitor in cell migration was relieved by sh-SDC3. (**F**) The promotion effect of miR-138-5p inhibitor on cell invasion was impaired by sh-SDC3. Blank, blank control. NC, negative control. Sh, sh-SDC3. Inhibitor, miR-138-5p inhibitor. *P<0.05, **P<0.001 compared with blank control. ^##^*P*<0.001 compared with co-transfection of sh-SDC3 and miR-138-5p inhibitor.

The transfection efficiency of sh-SDC3 and miR-138-5p inhibitor in OvC cells was detected before performing the cell-function experiments. Findings demonstrated that the transfection of sh-SDC3 and miR-138-5p inhibitor, respectively, reduced SDC3 expression and miR-138-5p expression by 70%. It was also found that miR-138-5p inhibition enhanced SDC3 expression by 2-fold; nonetheless, sh-SDC3 did not affect miR-138-5p expression (Figure 6A). The western blot assay results further confirmed that miR-138-5p inhibition elevated SDC3 protein level by more than 1.5-fold and that the SDC3 protein level was reduced by approximately 50% in the sh-SDC3 group (Figure 6B). Next, the result of the CCK8 assay, which was used to evaluate cell viability, indicated that sh-SDC3 impaired the cell viability at 48 and 72 hours; however, this inhibitory effect was neutralized by the promotive effect of the miR-138-5p inhibitor (Figure 6C). Similarly, the sh-SDC3 transfection inhibited colony formation and attenuated the promotive role of the miR-138-5p inhibitor on colony formation (Figure 6D). Experimental investigations also displayed that the cell migration rate was reduced by 60% in the cells transfected with sh-SDC3 compared to the blank group, while the cell migration rate of the sh-SDC3 and miR-138-5p inhibitor group was similar to that of the blank control group (Figure 6E). Similar to the cell migration assay result, the sh-SDC3 transfection inhibited cell invasion by more than 50%, and reversed the positive role of the miR-138-5p inhibitor on cell invasion (Figure 6F).

### Both cisplatin and sh-TRPM2-AS significantly promoted the apoptosis of OvC cells

**Figure 7:**
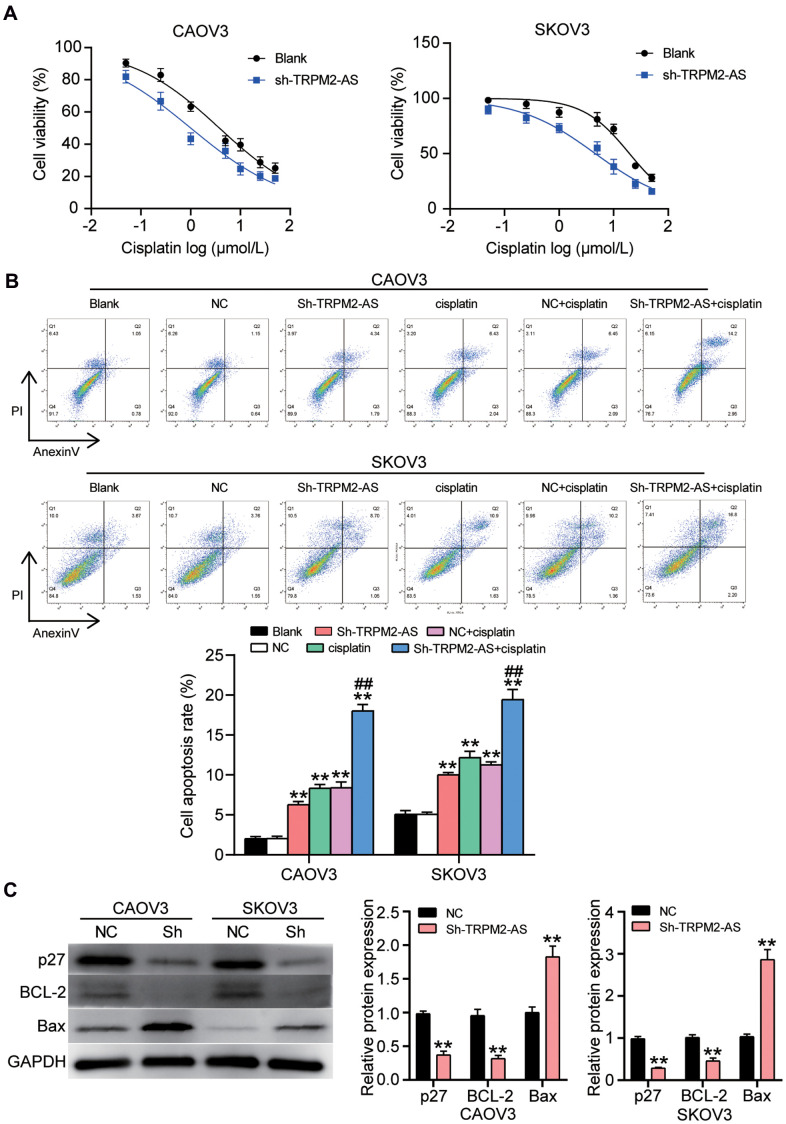
**The cell apoptosis could be enhanced by the combination of cisplatin and sh-TRPM2-AS in OvC cells.** (**A**) Cisplatin at corresponding IC50 concentrations inhibited 50% cell viability in CAOV3 and SKOV3 cells transfected with sh-TRPM2-AS. NC, negative control. (**B**) The combination of cisplatin and sh-TRPM2-AS significantly enhanced cell apoptosis in CAOV3 and SKOV3 cells. Blank, blank control. NC, negative control. **P<0.001 compared with blank control; ^##^*P*<0.001, compared with NC+cisplatin. (**C**) The OvC cells treated with cisplatin at corresponding IC50 concentrations and the transfection of sh-TRPM2-AS inhibited the protein expression of p27 and BCL-2, and promoted the protein expression of Bax. p27, BCL-2 and Bax were the cell apoptosis-associated proteins. NC, negative control. *P<0.05, **P<0.001 compared with negative control.

To identify the effect of TRPM2-AS on cisplatin resistance in OvC, we treated CAOV3 and SKOV3 cells transfected with sh-TRPM2-AS or negative control (NC) with various concentrations of cisplatin. The cell viability was detected using the CCK8 assay. The CCK8 assay results showed that the IC50 of CAOV3 and SKOV3 cells in the NC group treated with cisplatin was 3.78 μmol/L and 19.51 μmol/L, respectively. These values respectively decreased to 1.01 μmol/L and 4.61 μmol/L when TRPM2-AS was simultaneously silenced (Figure 7A). After that, the cell apoptosis rate was detected in the OvC cells transfected with sh-TRPM2-AS and simultaneously treated with cisplatin using the corresponding IC50 concentration. We observed that sh-TRPM2-AS significantly enhanced the apoptosis-promoting effect of the cisplatin (Figure 7B). We also detected the expression of the apoptosis-associated protein (p27, BCL-2 and Bax) in CAOV3 and SKOV3 cells with TRPM2-AS knockdown and treated with cisplatin (1.01 μmol/L for CAOV3 cell line and 4.61 μmol/L for SKOV3 cell line). The results displayed that TRPM2-AS knockdown and the simultaneous cisplatin treatment decreased the protein expression of p27 and BCL-2, and increased the expression of Bax by around 2-fold in OvC cells (Figure 7C).

## DISCUSSION

In our study, we identified the upregulation of TRPM2-AS and SDC3 in OvC tissues and cells. *In vitro*, TRPM2-AS contributed to cell proliferation, colony formation, cell migration and cell invasion. Moreover, TRPM2-AS knockdown was found to inhibit xenograft formation *in vivo*. We also found that TRPM2-AS could sponge miR-138-5p to release SDC3, thereby playing a tumor-promotive role in OvC cells. Besides, we noticed that cisplatin and sh-TRPM2-AS synergistically enhanced the apoptosis of OvC cells. Our findings revealed that the promotive effect of TRPM2-AS on OvC progression and cisplatin resistance, which was accomplished by sponging miR-138-5p to eliminate the inhibition of SDC3.

TRPM2-AS has been thought to influence the development of cancers. For example, one research reported that TRPM2-AS was associated with the poor prognosis of patients with gastric cancer and that the downregulation of TRPM2-AS suppressed the proliferation, metastasis and radioresistance of gastric cancer cells [27]. Another study used both co-expression network analysis (a bioinformatics method) and cell-function experiments to confirm that TRPM2-AS overexpression contributed to cell proliferation and inhibited cell apoptosis in breast cancer [13]. Ma et al. found that TRPM2-AS was overexpressed in A549 cells with cisplatin resistance and that TRPM2-AS knockdown could recover the cisplatin sensitivity in non-small cell lung cancer cells [16]. Based on the previous studies, we suspected that TRPM2-AS might be associated with the progression and cisplatin resistance of OvC. After performing cell-function experiments, we observed that TRPM2-AS played a tumor-promoter role in OvC cells shown by enhancing the proliferation and mobility phenotypes. Meanwhile, downregulated TRPM2-AS was discovered to impair the xenograft formation *in vivo*. As for cisplatin, we proved that silencing TRPM2-AS enhanced the apoptosis of OvC cells caused by 1 μmol/L cisplatin treatment. Besides, we also found that TRPM2-AS exerted a positive effect on OvC cells and cisplatin resistance by sponging miR-138-5p.

MiRNAs have been reported to influence the development of tumors by participating in biological processes [28–31]. One study on OvC found that miR-138 was downregulated in OvC tissues and that the low level of miR-138 in OvC patients was closely associated with lymphatic metastasis [20]. As for cisplatin resistance in OvC, miR-138-5p belonging to the miR-138 family was proved to enhance the cisplatin sensitivity of cisplatin-resistant cells, which could be attenuated by lncRNA HOTAIR [21]. Consistent with the previous study, our study indicated that the expression of miR-138-5p was reduced in OvC tissues and cells and that the downregulation of miR-138-5p enhanced the progression of OvC cells. Unlike the results of the previous study, we found that another lncRNA TRPM2-AS could sponge miR-138-5p to enhance the cisplatin resistance in OvC cells. We also confirmed that SDC3 was the target gene of miR-138-5p using luciferase reporter and RNA pull-down assays.

SDC3, a member of the syndecan proteoglycan family, is rarely reported, especially in cancer-related research. In their report that focused on the function of SDC3 in cancer, Yamada et al. [32] showed that the expression of SDC3 predicted the poor prognosis of patients with renal cell carcinoma and that SDC3 overexpression could reverse the negative effect of miR-144-5p on renal cell carcinoma cells due to their target relationship. In our study, we identified that SDC3 was the target gene of miR-138-5p and hypothesized that SDC3 might be an oncogene in OvC. After carrying out several experiments, we proved that SDC3 was overexpressed in OvC tissues and cells. We also demonstrated that silencing SDC3 by sh-SDC3 effectively inhibited the tumorigenesis of OvC cells, a result that revealed the promotive effect of SDC3 on OvC progression.

This research has several limitations. Although our study revealed the function of the TRPM2-AS/miR-138-5p/SDC3 axis in OvC tumorigenesis and cisplatin resistance, these findings might only be applicable for the pathogenesis of high-grade serous ovarian cancer (HGSC). Besides, how TRPM2-AS regulates other types of OvC progression and cisplatin resistance remains to be explored with the cell and mice models used in this study. The downstream regulatory mechanism of SDC3 in OvC and the role of the TRPM2-AS/miR-138-5p/SDC3 axis in cisplatin-resistant OvC cells also need to be investigated. These key regulatory mechanisms will be further researched in our future studies.

## CONCLUSION

Overall, this research demonstrated the role of the TRPM2-AS/miR-138-5p/SDC3 axis on the progression of OvC and the effect of TRPM2-AS on cisplatin resistance of OvC. Specifically, we found that TRPM2-AS could promote OvC progression by sponging miR-138-5p to release SDC3. We also noticed that TRPM2-AS knockdown could reduce the cisplatin resistance in OvC cells. Therefore, our research might be useful in unraveling novel therapies and treatments for OvC.

## MATERIALS AND METHODS

### Sample collection

A total of 42 paired OvC tissues and contralateral normal fallopian tube tissues were collected from 42 OvC patients who underwent tumor excision at Tongji Hospital. The tumor excision and tissue collection were performed after the patients were diagnosed with OvC prior to first-line chemotherapy. After that, normal fallopian tube tissues were collected from 16 patients with benign gynecological tumors, who underwent salpingo-oophorectomy at Tongji Hospital, and these samples were used as the normal controls. The experimental procedure was approved by the Ethics Committee of Tongji Hospital. Before the experiments commenced, all the participants (42 OvC patients and 16 patients with benign gynecological tumors) were asked to complete the informed consent forms. The clinical characteristics of 42 OvC patients are shown in Table 1.

**Table 1:** Clinical characteristics of 42 cases of ovarian carcinoma patients.

**Characteristics**	**Total = 42**	**Percentage (%)**
Age(years)
>60	19	45.2%
≤60	23	54.8%
Tumor histological type ^a^
HGSC	28	66.7%
LGSC	3	7.1%
EC	5	11.9%
CCC	6	14.3%
Tumor stage
I	3	7.1%
II	4	9.5%
III	27	64.3%
IV	8	19.0%
Lymph node metastases
Negative	14	33.3%
Positive	28	66.7%

^a^, 2014 WHO classification criteria of ovarian cancer. HGSC, high-grade serous carcinoma; LGSC, low-grade serous carcinoma; EC, endometrioid carcinoma; CCC, clear cell carcinoma.

### Detection of lncRNA, miRNA and mRNA expression

The expression of lncRNA (TRPM2-AS), miRNA (miR-138-5p) and mRNA (SDC3 mRNA) was detected using qRT-PCR. Briefly, the total RNAs were isolated from tissues and cells with TRIzol Reagent (Cat#: 15596018, Invitrogen, USA). After isolating the RNAs, the PrimeScript RT Reagent Kit (Cat#: RR037Q, TaKaRa, Japan) was employed to synthesize the cDNA. Finally, a qRT-PCR analysis was performed using SYBR Premix Ex Taq II (Cat#: DRR820A, TaKaRa, Japan) and SYBR Green Supermix (BIO-RAD, USA). The reference gene for TRPM2-AS and SDC3 was GAPDH, while the reference miRNA for miR-138-5p was U6. Subsequently, the localization of TRPM2-AS was evaluated using the Cytoplasmic and Nuclear RNA Purification Kit (Invitrogen, USA). The enrichment of TRPM2-AS in the cytoplasm and nucleus was then identified using qRT-PCR. All the primer sequences used in this study are listed in Table 2.

**Table 2:** The primer sequences for RT-qPCR.

**Gene**	**Primer sequences (5’-3’)**
TRPM2-AS	Forward: CCAGGAACCAGAACCAAACT
	Reverse: TGTCCGTCTGCTGAGACATC
SDC3	Forward: GACTCCTTTCCCGATGATGA
	Reverse: GTCAGTGGGAGAGGCAGAAG
GAPDH	Forward: TGCACCACCAACTGCTTAGC
	Reverse: GGCATGGACTGTGGTCATGAG
miR-138-5p	Forward: AGCTGGTGTTGTGAATCAGGCCG
	Reverse: GCGAGCACAGAATTAATACGAC
U6	Forward: CTCGCTTCGGCAGCACA
	Reverse: AACGCTTCACGAATTTGCGT

### Cell culture and transfection

Human ovarian surface epithelial cell line IOSE-80 and four OvC cell lines (EB0405, CAOV3, HEY and SKOV3) used in this study were purchased from the National Infrastructure of Cell Line Resource (China). IOSE-80 cells were kept in the RPMI-1640 medium (Gibco, USA), which was supplemented with 10% fetal calf serum (FBS, Gibco, USA). The OvC cell lines, on the other hand, were kept in the DMEM medium (Gibco, USA), which was supplemented with 10% FBS. After the cell confluence reached 50%, the cells were transfected with sh-TRPM2-AS, TRPM2-AS OE (overexpression plasmids), miR-138-5p inhibitor, sh-SDC3 and the corresponding negative control vectors (NC). This transfection procedure was done with the Lipofectamine 3000 Transfection Reagent (Invitrogen, USA). All the vectors were designed and constructed by Shanghai OBIO Technology Corp., Ltd. (China). Specifically, the TRPM2-AS overexpression plasmids was constructed by cloning full length TRPM2-AS sequence into pcDNA3.1 plasmid. The transfection efficiency of these vectors was confirmed using qRT-PCR. The sequences of sh-TRPM2-AS and sh-SDC3 are illustrated in Table 3.

**Table 3:** The clone sequences in this study.

**Name**	**Sequences (5’-3’)**
sh-TRPM2-AS	Sense: GGGAAGATGTCTCAGCAGA
	Anti-sense: TCTGCTGAGACATCTTCCC
sh-SDC3	Sense: GACACGTACTGCAATGCAA
	Anti-sense: TTGCATTGCAGTACGTGTC

### The detection of cell viability

A CCK8 assay was employed to assess the cell viability using Cell Counting Kit-8 (CCK-8) (Dojindo, Japan). This assessment was done according to the manufacturer’s protocol. That is, 5×10^3^ cells/well were seeded into 96-well plates and incubated overnight. Then, the transfected cells were cultured for 0, 24, 48 or 72 hours, and the CCK-8 solution (10 μL/well) was then added to the cells. After incubating the cells for another 2 hours, the absorbance value was measured at 450 nm with a microplate reader.

### Colony formation assay

This assay was used to assess the number of formed colonies or the level of cell proliferation. Briefly, the transfected cells were seeded into 6-well plates at a density of 500 cells/well and then incubated. The medium was subsequently replaced with a fresh medium every day for two weeks. Two weeks later, 4% paraformaldehyde and 0.1% crystal violet solution were used to fix and stain the cells, respectively. Finally, the number of formed colonies was photographed and counted under a microscope.

### Wound healing assay

The wound-healing assay was performed to evaluate cell migration. The cells transfected (1×10^5^) were first seeded into 6-well plates and then incubated. When the cell confluence reached 90%, the medium containing 10% FBS was replaced with a serum-free medium and then subjected to 12-hour serum-starvation treatments. Next, the cell monolayers were scratched with the tips of 200-μL pipettes, and the exfoliated cells were washed off with PBS. Finally, the cells were cultured with the fresh medium for 24 hours, and the cell migratory images were obtained with a microscope. The migration rate was calculated as the ratio of the difference of the gap distances at 0 h and 24 h to the gap distance at 0 h.

### Detection of cell invasion

Transwell invasion assay was conducted to assess the cell invasion. Briefly, 1×10^5^ cells transfected for 48 hours were seeded into the upper chamber of the cell well. The 600 μL DMEM medium containing 10% FBS was added to the lower chamber of the cell well. After 24 hours, the cells on the upside of the membrane were removed with cotton swabs, while the cells on the downside membrane were fixed with 4% paraformaldehyde. After the cells were stained with 0.1% crystal violet, the invasive cells were observed and photographed with a microscope.

### Xenograft formation assay

A total of ten female BALB/C nude mice obtained from Charles River Labs (China) were randomly divided into two groups: the NC group and the sh-TRPM2-AS group. Next, 2×10^5^ SKOV3 cells transfected with sh-NC or sh-TRPM2-AS were subcutaneously respectively injected into the flanks of the mice in the NC group and the sh-TRPM2-AS group. After 30 days, the mice were sacrificed to remove the tumors. The tumor volume (mm^3^) was calculated with the following formula: V (mm^3^) = L (mm) × W^2^ (mm^2^). L and W represented tumor length and tumor width, respectively.

### Immunohistochemistry (IHC)

The sections (thickness of 5 μm) from human OvC tissues or mice tumor tissues were dewaxed with xylene and hydrated with gradient alcohol. Next, the endogenous peroxidase in the tissue sections was inactivated by the peroxide blocker. The sections were then incubated with the primary antibody against SDC3 (Cat#: ab36653, Abcam, China) or primary antibody against Ki67 (Cat#: ab15580, Abcam, USA). This step was followed by the incubation of the samples with anti-rabbit IgG (Cat#: ab6721, Abcam, USA). DAB-Peroxidase Substrate Solution (Servicebio, Wuhan, China) was subsequently used to change the positive cells to brown. After the sections were counterstained with hematoxylin solution, the images of IHC were captured with a microscope.

### Hematoxylin and eosin (H&E) staining

The sections from the human OvC tissues or mice tumor tissues were first dewaxed with xylene and then hydrated with gradient alcohol. After that, hematoxylin staining was performed for 3 minutes. Next, the stained tissue sections were treated with 1% hydrochloric acid alcohol for 20 seconds. After the sections were stained with eosin for 5~10 seconds, they were dehydrated and mounted with neutral balsam. The images were finally taken with a microscope.

### Luciferase assay

ENCORI starBase and TargetScan were employed to predict the binding sites between TRPM2-AS and miR-138-5p, and between SDC3 mRNA 3’UTR and miR-138-5p, respectively. The wild-type (WT) TRPM2-AS, mutant (MUT) TRPM2-AS without the binding site and WT SDC3 mRNA 3’UTR were cloned into the psiCHECK2 vectors. Because of the two binding sites of SDC3 mRNA 3’UTR for miR-138-5p, the single or co-mutation of two binding sites was cloned into the psiCHECK2 vectors such as Mut1-SDC3, Mut2-SDC3, and Co-mut-SDC3. These WT or MUT constructs were then co-transfected into CAOV3 and SKOV3 cells with miR-138-5p mimic or negative control (miR-NC). After co-transfecting for 48 hours, the dual-luciferase reporter assay system (Promega, USA) was used to detect the luciferase activity.

### RNA immunoprecipitation (RIP) assay

The Magna RIP RNA-Binding Protein Immunoprecipitation Kit (Millipore, USA) was utilized to perform the RIP assay and confirm the binding relationship between TRPM2-AS and miR-138-5p. AGO2 or IgG (control), the antibody used for this assay, was purchased from Millipore (USA). Before the RIP assay was performed, the CAOV3 and SKOV3 cells were transfected with the negative control or miR-138-5p mimic for 48 hours. Then, the transfected cells were collected and centrifuged at 1500 rpm for 5 minutes. After discarding the supernatant, the cells were lysed with the RIP lysis buffer, and the magnetic beads conjugated to AGO2 or IgG was added to the lysate in 900 μL RIP Immunoprecipitation buffer containing 35 μL of 0.5 M EDTA and 5 μL RNase inhibitor. After the RNA was purified by adding proteinase K, it was isolated with TRIzol Reagent. The enrichment of TRPM2-AS was finally detected using qRT-PCR.

### RNA pull-down assay

The RNA 3’ End Desthiobiotinylation Kit (Pierce, USA) was employed to perform this assay to confirm the binding site between SDC3 mRNA 3’UTR and miR-138-5p. The biotin-labeled negative control (Bio-NC) and biotin-labeled miR-138-5p (Bio-miR-138-5p) were constructed and provided by Shanghai OBIO Technology Corp., Ltd. (China). The cells transfected with Bio-NC or Bio-miR-138-5p were lysed and incubated with streptavidin magnetic beads. After adding protein K and DNase A, the RNA was isolated, and the SDC3 mRNA pulled down was detected using qRT-PCR.

### Western blot analysis

The total protein from the cells was isolated using a lysis buffer with protease inhibitors. Then, the isolated protein was separated on 12% SDS-PAGE gels, followed by an electrophoretic transfer to PVDF membranes (Millipore, USA). After blocking the membranes in 5% nonfat milk, they were incubated overnight with primary antibodies against SDC3 (Cat#: ab155952, Abcam, USA), p27 (Cat#: ab32034, Abcam, USA), BCL-2 (Cat#: ab32124, Abcam, USA), Bax (Cat#: ab32503, Abcam, USA) and GAPDH (Cat#: ab9485, Abcam, USA). The next day, the anti-rabbit IgG (Cat#: ab6721, Abcam, USA) was incubated with the membranes for 3 hours. Finally, the protein bands were detected using the ChemiDoc Imaging System (BIO-RAD, USA). The intensities of the bands were analyzed using Image J software.

### Cell apoptosis detection

Flow cytometry was used to assess the rate of cell apoptosis. This assessment was done with the V-FITC Apoptosis Detection Kit (Invitrogen, USA). The 1×10^6^ CAOV3 and SKOV3 cells transfected with sh-TRPM2-AS and/or treated with cisplatin were collected and washed in PBS. Then, the cells were resuspended in 200 μL binding buffer (1×). After that, 5 μL Annexin V-FITC and 10 μL Propidium Iodide (PI, 20 μg/mL) were added to the cell suspension, and the mixture was incubated in the dark for 15 min. Lastly, the apoptosis was analysed using flow cytometry.

### Cisplatin IC50 detection

For the detection of cisplatin IC50, 5×10^3^ CAOV3 and SKOV3 cells were first seeded into each well of 96-well plates and incubated overnight. Then the cells were treated with gradient concentrations of cisplatin for 48 h, which included 0.05, 0.25, 1, 5, 10, 20 and 50 μmol/L. Afterwards, the cell viability was measured by CCK-8 assay, and the cisplatin IC50 of the two cell lines was calculated with GraphPad Prism 8.0.

### Statistical analysis

All the data used in this study were obtained from at least three independent experiments, and were presented in the form of mean ± standard deviation (SD). GraphPad Prism 8.0 was utilized to analyze the data. The Student’s *t*-test was used to compare the statistical difference between two groups, while the one-way ANOVA was used to compare the statistical differences between multiple groups. Only p-values of less than 0.05 were considered statistically significant.

### Availability of data and materials

The data used and analyzed during the current study are available from the corresponding author on reasonable request.

### Ethics approval and consent to participate

The study was approved by the Ethics Committee of Tongji Hospital. All procedures meet ethical standards set out in the Helsinki Declaration. All patients signed written informed consent. The approval NO. for the clinical study is [2020] No. (S229), and for animal experiments is [2020] No. (S2373) ([2020] IACUC NO. 2373).
